# Telomere Reprogramming and Maintenance in Porcine iPS Cells

**DOI:** 10.1371/journal.pone.0074202

**Published:** 2013-09-30

**Authors:** Guangzhen Ji, Weimin Ruan, Kai Liu, Fang Wang, Despoina Sakellariou, Jijun Chen, Yang Yang, Maja Okuka, Jianyong Han, Zhonghua Liu, Liangxue Lai, Sarantis Gagos, Lei Xiao, Hongkui Deng, Ning Li, Lin Liu

**Affiliations:** 1 State Key Laboratory of Medicinal Chemical Biology, College of Life Sciences, Nankai University, Tianjin, China; 2 State Key Laboratory for Agrobiotechnology, College of Biological Sciences, China Agricultural University, Beijing, China; 3 Laboratory of Genetics, Center of Basic Research II, Biomedical Research Foundation of the Academy of Athens Greece (BRFAA), Athens, Greece; 4 College of Animal Sciences, Stem Cell and Developmental Biology Research Center, Zhejiang University, Hangzhou, Zhejiang, China; 5 College of Life Sciences, Peking University, Beijing, China; 6 Department of Obstetrics and Gynecology, University of South Florida College of Medicine, Tampa, Florida, United States of America; 7 Life Science College, North-east Agricultural University, Harbin, Heilongjiang, China; 8 Key Laboratory of Regenerative Biology, Guangzhou Institutes of Biomedicine and Health, Chinese Academy of Sciences, Guangzhou, Guangdong, China; University of Science and Technology of China, China

## Abstract

Telomere reprogramming and silencing of exogenous genes have been demonstrated in mouse and human induced pluripotent stem cells (iPS cells). Pigs have the potential to provide xenotransplant for humans, and to model and test human diseases. We investigated the telomere length and maintenance in porcine iPS cells generated and cultured under various conditions. Telomere lengths vary among different porcine iPS cell lines, some with telomere elongation and maintenance, and others telomere shortening. Porcine iPS cells with sufficient telomere length maintenance show the ability to differentiate *in vivo* by teratoma formation test. IPS cells with short or dysfunctional telomeres exhibit reduced ability to form teratomas. Moreover, insufficient telomerase and incomplete telomere reprogramming and/or maintenance link to sustained activation of exogenous genes in porcine iPS cells. In contrast, porcine iPS cells with reduced expression of exogenous genes or partial exogene silencing exhibit insufficient activation of endogenous pluripotent genes and telomerase genes, accompanied by telomere shortening with increasing passages. Moreover, telomere doublets, telomere sister chromatid exchanges and t-circles that presumably are involved in telomere lengthening by recombination also are found in porcine iPS cells. These data suggest that both telomerase-dependent and telomerase-independent mechanisms are involved in telomere reprogramming during induction and passages of porcine iPS cells, but these are insufficient, resulting in increased telomere damage and shortening, and chromosomal instability. Active exogenes might compensate for insufficient activation of endogenous genes and incomplete telomere reprogramming and maintenance of porcine iPS cells. Further understanding of telomere reprogramming and maintenance may help improve the quality of porcine iPS cells.

## Introduction

IPS technology provides great potential for therapeutic uses, modeling human diseases and drug discovery [1], [2]. The pig has been frequently noted as a superior biologically relevant model, with anatomy and physiology comparable to humans [3], [4], and also provides appropriate xeno-transplantation sources and a model for study of human diseases [5]–[8]. Generation of porcine iPS cells complements studies of human iPS cells [9], [10], as the safety and effectiveness of iPS cells for therapeutics not only can be evaluated by genomic and epigenomics, but also can be functionally assessed by cell transplantation [11], and tested by germline chimeras in pigs. Porcine iPS cells show self-renewal and pluripotency by expression of pluripotent genes and differentiation into three embryonic germ layers *in vitro* by teratoma formation [12]–[18]. Moreover, porcine iPS cells can generate chimeras with germline competence, further proving their pluripotency [19], [20], and recently produce cloned piglets [21].

Telomere length maintenance and homeostasis are essential for unlimited self-renewal and pluripotency of ES and iPS cells [22], [23]. Telomeres consist of repeated TTAGGG sequences and associated proteins at the chromosome ends that maintain chromosomal and genomic stability [24], [25]. Telomere lengths are maintained primarily by telomerase [26], [27]. Three major components, TERT, TERC, and dyskerin, determine telomerase activity [28]–[31]. Telomeres can be effectively reprogrammed and exo-transgenes are silenced in mouse and human induced pluripotent stem (iPS) cells, despite that telomere length varies in various iPS cell lines [32]–[36]. Notably, most of porcine iPS cells generated in many laboratories exhibit activated exogenes (or exogenous transcription factors) or incomplete silencing of exogenes [12]–[16], [19], unlike complete silencing of exogenes in mouse and human iPS cells [37], [38]. It remains unclear whether porcine iPS cells acquire effective telomere reprogramming and maintenance. Hence, we performed systematic analysis of telomere length and maintenance in various porcine iPS cells generated by various methods from several cell types. We found that telomere reprogramming occurs during porcine iPS induction and telomere lengths vary among different porcine iPS cells and that incomplete telomere reprogramming and maintenance are associated with active exogenous genes in porcine iPS cells.

## Results

### Porcine iPS cells generated by various methods from different cell types

**Figure 1:**
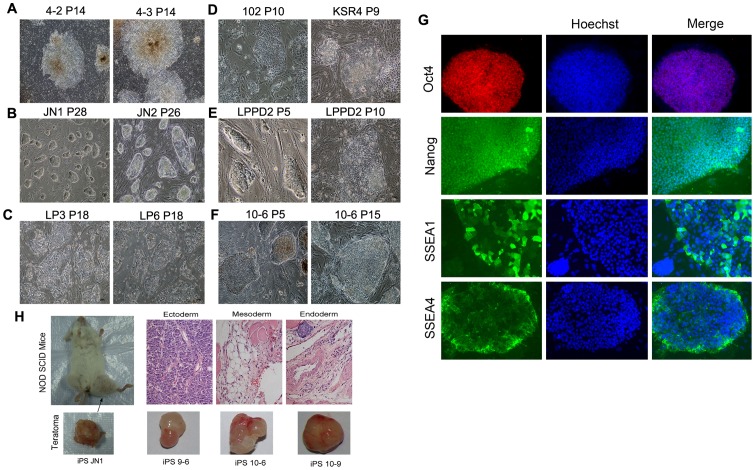
Characterization of porcine iPS cells. (A–F) Typical morphology of various pig iPS cell lines. (A) Human ES-like pig iPS that induced by human factors and cultured in human ES-like medium. The iPS colonies are large and flat. The colony is less compact than human ES cells and the edge of the colony is irregular. (B) Mouse ES-like pig iPS induced by mouse factors and cultured in human- mouse ES culture medium. (C) iPS morphology of LP cell lines. The colonies at late passages become incompact and some cells differentiated, though nuclear-cytoplasm ratio is clearly seen. (D) Morphology of exo-genes-silenced pig iPS cell lines. The 102 iPS cells start to differentiate at passage 10. Incompact and domed colonies co-exist in KSR4P9. (E) Morphology of pig iPS derived and cultured with small molecules. The colonies at early passage (LPPD2P5) are domed and show clear nuclear-cytoplasm ratio, however, colonies become flattened and begin to differentiate at later passages. (F) Morphology of iPS 10–6 generated from PEF using porcine factors and cultured in human ES medium. Nice colonies like human ES still maintained by passage 15. (G) Representative images showing expression of pluripotent markers Oct4, Nanog, SSEA4, and SSEA1 of iPS 10–6 by immunofluorescence staining and microscopy. Cells without the first antibody incubation served as the negative control. (H) Teratomas formed from pig iPS cells. Shown are three typical germ layers by histological section of teratomas from iPS JN1, and teratomas from iPS 9 and iPS 10 (weight around 0.5–1.4 g).

Various porcine iPS cells (detailed in Table 1) were employed for investigation of telomere rejuvenation. Morphologically, porcine iPS cells exhibited doom-like colonies like mouse ES/iPS cells, when cultured under mouse ES culture conditions, whereas they showed flattened clones like human ES/iPS cells, when cultured under human ES culture conditions (Figure 1A–F). Dox-inducible, lentivirus-mediated transduction of human Oct4, Sox2, cMyc and Klf4 resulted in high efficient generation of porcine iPS cells [12]. The *in vivo* teratoma formation assay is commonly used as the “gold standard” to test pluripotent capacity of human ES/iPS cells [39], [40]. The iPS cells iPS4-2 and iPS4-3 induced from porcine fetal fibroblasts (PFX), expressed pluripotency related genes, and generated three germ layers by teratoma test [12] (Table 1). Porcine iPS cells also were generated from porcine newborn mesenchymal cells isolated from bone marrow (NM) or embryonic fibroblasts by retrovirus-transduced expression of mouse Yamanaka factors. All iPS cell lines were positive for alkaline phosphatase (AP) activity and also expressed pluripotency-associated marker genes by immunofluorescence (Figure 1G and Table 1), including Oct3/4, Nanog, and SSEA3/4. Porcine iPS cells JN1 at early passages could form teratomas and differentiate into cell types of three germ layers *in vitro* (Figure 1H, Table 1). Also, iPS cells 9–6 and 10–6 and 10–9 derived using porcine factors efficiently formed teratomas (weight ranging from 0.5 g to 1.4 g) within one month following injection into immunodeficient mice (Figure 1H, Table 1). However, other porcine iPS cells LP3 and LP6 expressed pluripotent genes but failed to pass the teratoma tests, thus were less pluripotent (Table 1).

**Table 1:** Various porcine iPS cells analyzed in this study.

iPS Cell line	Primary cell	Infected Factors	Culture condition	AP staining	Pluripotent Gene Expression						Exo-gene Silence	Teratoma
					Oct4	Nanog	SSEA1	SSEA3/4	TRA-1-60	TRA-1-80		
4–2	PFX	hOSKM	hES medium*	+	+	+	NA	+	+	+	−	+
4–3	PFX	hOSKM	hES medium*	+	+	+	NA	+	+	+	−	+
JN1	NM	mOSKM	hES+mES I*	+	+	+	+/−	+	+	+	−	+/−*
JN2	NM	mOSKM	hES+mES I*	+	+	+	+/−	+	+	+	−	−
LP3	SWF	hOSKM	hES+mES II*	+	+	+	+/−	+	+	+	−	−
LP6	SWF	hOSKM	hES+mES II*	+	+	+	+/−	+	+	+	−	−
68	LFF	mkOSKM	hES+mES II*	+	+	+	NA	+	+	+	+	+
102	LFF	mkOSKM	hES+mES II*	+	+	+	NA	+	+	+	+	+
KSR4	LFF	pOSKM	mES medium	+	+	+	NA	NA	NA	NA	+	−
LPPD2	PEF	pOSKM	mES medium	+	+	+	NA	NA	NA	NA	−	+
CHH	HH	pOSKM	mES medium	+	+	+	NA	NA	NA	NA	−	−
9–6 (16)	PEFL	pOSKM	hES medium#	+	+	+	+/−	+	NA	NA	−	+
10–6	PEFL	pOSKMN	hES medium#	+	+	+	+/−	+	NA	NA	−	+
10–9	PEFL	pOSKMN	hES medium#	+	+	+	+/−	+	NA	NA	−	+

Primary cells or progenitor cells: PFX, newborn porcine ear fibroblast; NM, mesenchymal cells form new born porcine bone marrow; SWF, embryonic porcine fibroblast; LFF, embryonic porcine fibroblast (Taihu breed); PEF, porcine embryonic fibroblast (Yorkshire breed); HH, adult pig era fibroblast (Yorkshire breed); PEFL, porcine embryonic fibroblast from Nong Da Xiang mini-pig. Human OSKM (hOSKM), human Oct4, Sox2, Klf4 and Myc; mouse OSKM (mOSKM), mouse Oct4, Sox2, Klf4 and Myc; porcine OSKM (pOSKM), porcine Oct4, Sox2, Klf4 and Myc or pOSKMN (N for Nanog); monkey OSKM (mkOSKM), monkey Oct4, Sox2, Klf4 and Myc. hES medium*, DMEM/F12 added with 20% KSR; hES+mES I, Knockout DMEM added with 20%FBS, 4 ng/mL bFGF, 1000U/mL mLif; hES+mES II, DMEM/F12 added with 10% KSR, 10% FBS, 2 ng/mL bFGF, 500U/mL Lif; hES medium II**, DMEM/F12 added with 20% KSR, and 4 ng/ml bFGF; mES medium, Knockout DMEM added with 20% KSR, 10 ng/mL hLif, and some added with small molecules (PD0325901/CHIR99021, PD and CH). hES medium#, Knockout DMEM added with 20%KSR and 10 ng/mL bFGF. NA, data not available.

Furthermore, the quality and pluripotency of the porcine iPS cells was assessed by nuclear transfer test. Porcine iPS4-2 successfully generated live cloned piglets by nuclear transfer technique, and several cell lines demonstrated their fetal development to gestation of day 30–90 days but without live-born offspring by nuclear transfer [21], and by chimera production. These data suggest that some porcine iPS cells meet the criteria for pluripotency by teratoma formation test, but still remain incompetent in generation of germline chimeras.

### Exogenous gene silencing and activation of endogenous genes

Various methods can generate porcine iPS cells with differentiation potential into three germ layers by teratoma tests, but notably, exogenes were not silenced and remained active in these cells [12], [13], [15]. Porcine iPS 4–2 exhibited reduced expression of exogenes following removal of Dox, but expression of pluripotency genes also declined, suggesting that maintenance of pluripotency requires sustained expression of exogenes [12]. iPS 4–2 and JN1 maintained relatively stable expression of pluripotency genes Oct4, Sox2, Klf4, Myc and Nanog [12] (Figures S1A and S2). iPS cells JN1/2 still exhibited high expression levels of exogenes, but reduced compared with the controls (Figure S1B and Table S1). iPS cells LP3 showed reduced expression of pluripotent genes Nanog, Sox2, Klf4 and Myc with increasing passages (Figure S2B). These data further suggest that maintenance of porcine iPS cells requires sustained active exogenes [15].

Cultures in KSR, or under low oxygen, and addition of MAPK and GSK3 inhibitors were shown to improve iPS generation and ES naïve pluripotency state [41]–[44]. We assumed that exogene silencing might be enhanced by clonal selections, change of culture conditions, or inhibition of MAP kinase. We attempted to select as many clones as possible, and cultured cells under different conditions. Kenpaullone, GSK3β inhibitor, and SB431542, a TGF-β inhibitor, indeed reduced expression of exogenes and enhanced expression of endogenous Nanog, Sox2 and Klf4, but not Oct4, Myc and Lin28 (Figure S1C). By subculture of actively selected clones, we were able to generate porcine iPS cells with silencing or partial silencing of exogenous genes (Table 1). Three iPS cell lines exhibited silencing or partial silencing of exogenous transcriptional factors. Others still required active exogenes to maintain their self-renewal (Table 1 and Figures S2C and S2D). iPS KSR4 showed exogene silencing at early passages (P5) and activation of endogenous Oct4 and Nanog, but reactivation of exogenes with passages (P9-10), accompanied by reduced expression of Oct4 and Nanog (Figure S2C). iPS cells 68 and 102 with exogene silencing expressed pluripotent marker genes and also formed teratomas at early passages, but at reduced rates, compared to those with active exogenous genes, and tended to differentiate during longer-term culture. However, iPS LPPD2 cells induced and cultured under low oxygen and inhibition of MAPK still did not show silencing of exogenous genes except for exo-Sox2 at early passage P5, but efficiently generated teratomas (Figure S2D and Table 1).

### Various telomere dynamics of porcine iPS cells

**Figure 2:**
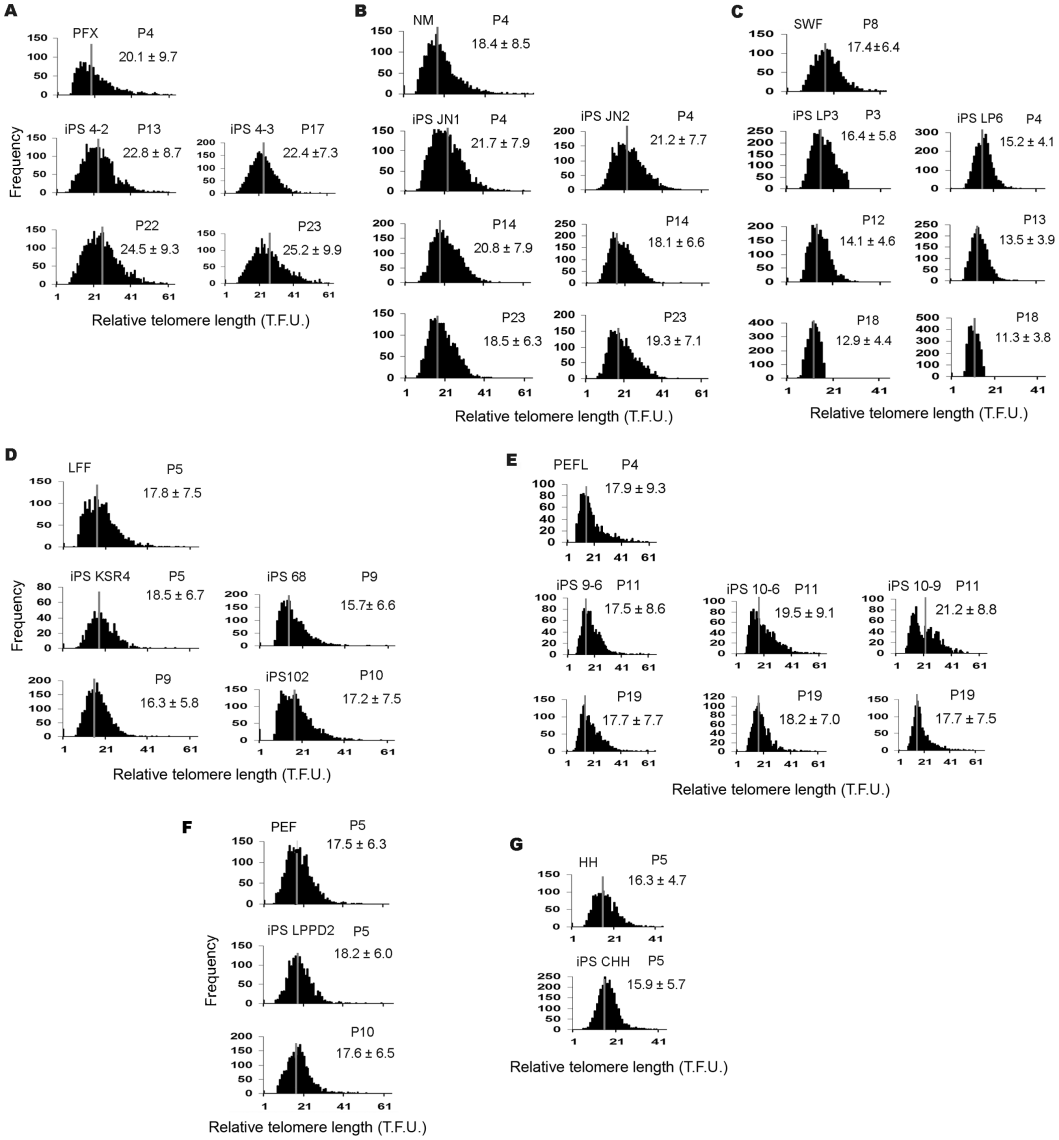
Telomere relative length and distribution estimated by telomere quantitative fluorescence *in situ* hybridization (Q-FISH). (A–B) Telomere lengths increased after iPS generation and maintained in iPS cell lines (iPS4-2 or 4–3) that exhibited teratoma formation and stable proliferation. Telomeres were slightly elongated in iPS JN1/2 during induction but not maintained during passages. (C) Relative telomere lengths decreased in LP cell lines that failed to form terotoma. (D) Telomere length shortened in iPS cell lines with exo-gene silence. (E) Telomere maintenance in iPS9 and iPS10 generated using porcine factors and by culture under human ES culture condition. These iPS cells exhibit teratoma formation. (F and G) Telomere length in pig iPS cells induced by small molecules and low oxygen was similar to that of the progenitors. 1 T.F.U approximates 1.26 kb. Histograms show distribution of relative telomere length as fluorescence intensity (T.F.U., telomere fluorescence unit) from one cell line of each group. Heavy black bars on Y-axis indicate number of telomere signal-free ends (detailed in Figure S5). Gray line indicates average telomere length (T.F.U.). Average telomere length as TFU is indicated at upper right hand corner (Mean ± Sd).

**Figure 3:**
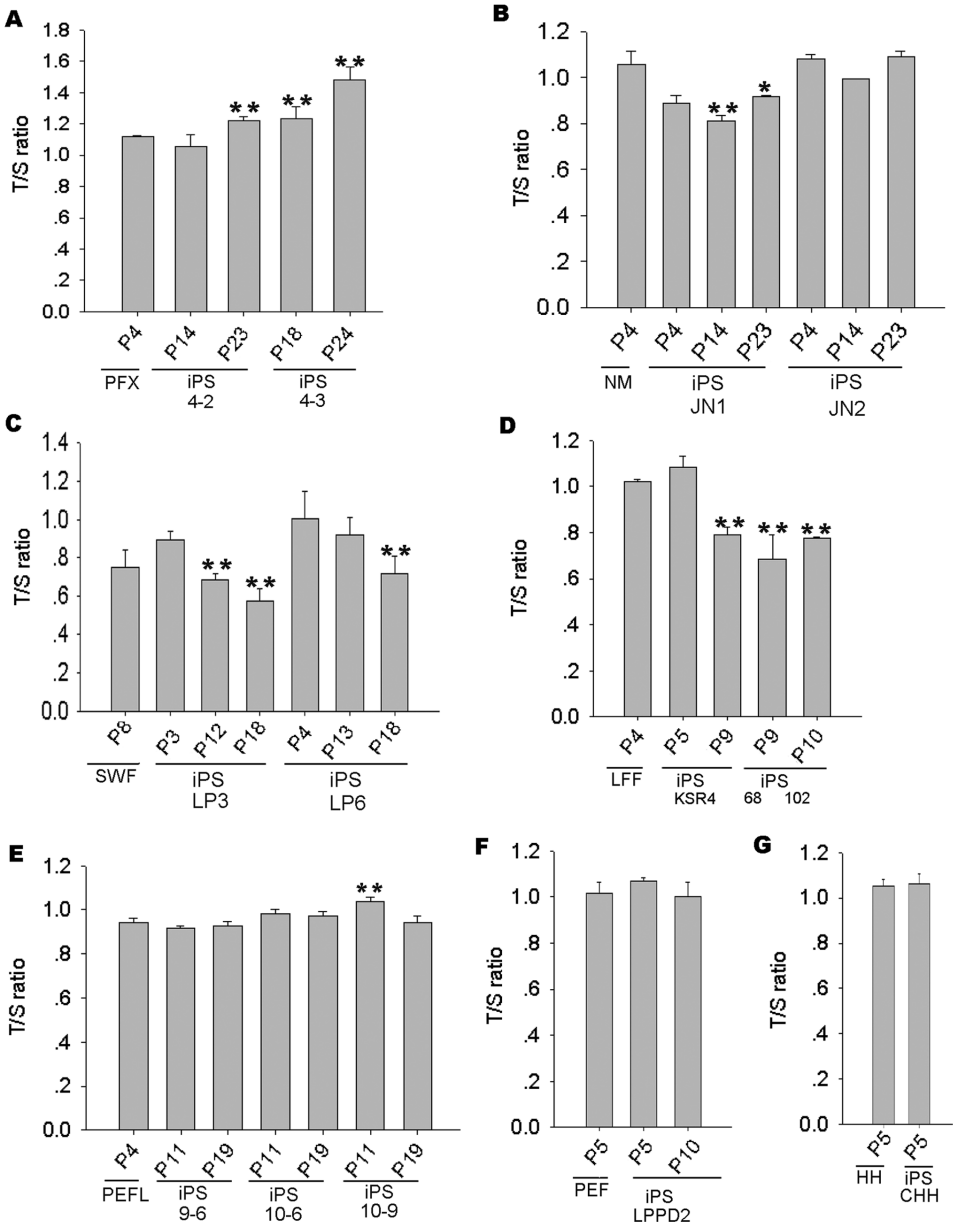
Telomere length dynamics at various passages measured by quantitative real-time PCR analysis (qPCR). Relative telomere length is shown as T/S ratios. T, telomere; S, 36B4 single-copy gene. (A) Telomere length increased during subculture. (B) Telomere length increased then decreased or became relatively unstable. (C) Telomere length decreased during passages. (D) iPS with exo-gene silencing showed shorter telomeres compared with progenitor cells. (E) Telomere length maintained in early passage iPS cells. (F, G) Telomere length did not differ between iPS cells and their progenitors. PFXP4, NMP4, SWFP8, LFFP4, PEFL, PEFP5 and HHP5 were primary progenitor cells, respectively. P, passage. *, p<0.05; **, p<0.01, compared to primary cells or progenitor cells. (n = 3 independent replicate).

We measured telomere lengths in various iPS cells at different passages and found that dynamics of telomeres differed among the cell lines. Telomeres lengthened in the porcine iPS cell lines 4–2 and 4–3 at early passages (P13), and continued elongation by passage 22–23, compared to their progenitor cells (PFX P4) (Figure 2A). Notably, these iPS cells efficiently formed teratomas. Telomere length maintained or slightly elongated in porcine iPS cells JN1 at early passages compared to their progenitors NMP4, coincided with their ability to form teratomas, but shortened over the passages (Figure 2B). Telomere length measurement by Q-FISH was roughly consistent with the data measured by qPCR (Figure 3A and 3B). Porcine iPS cells LP3 and LP6 maintained telomeres at earliest passages (P3), but their telomeres shortened with increasing passages by Q-FISH (Figure 2C). The slightly elongated telomeres of these cells became shorter with the passages, shown by qPCR measurement (Figure 3C). Notably, telomeres slightly elongated in iPS KSR4 at early passage (P5) with silenced exogenous genes, but shortened with increasing passages (Figures 2D and 3D). Also, iPS cell lines 68 and 102 with exogene silencing showed telomere shortening with passages (Table 1). These iPS cells formed teretomas at early passages, but less efficiently. The iPS cells 9–6 and 10–6 and 10–9 maintained telomeres by passage 10–11, and showed only slight telomere changes after additional eight passages (Figures 2E and 3E). Telomere lengths were maintained in porcine iPS cells when small molecules such as PD (ERK1/2 inhibitor PD0325901), CH (GSK3 inhibitor CHIR99021) and Kenpaullone, were added in the culture medium for limited passages (Figures 2F,2G and 3F,3G). However, these iPS cells failed to maintain telomeres following prolonged cultures and underwent limited proliferation (data not shown). Thus, telomere reprogramming differed in various porcine iPS cells compared to their primary progenitor cells, as shown by two independent telomere length measurement methods (qPCR and Q-FISH). Together, these data suggest that telomeres can be elongated and maintained in the porcine iPS cells tested particularly when exogenous genes remain active, whereas telomere elongation is limited and telomeres tend to shorten in porcine iPS cells when exogenous genes are silenced and endogenous genes are not fully activated. Telomere lengths appear to be important for pluripotency by teratoma tests. IPS cells with telomere shortening failed to form teratomas, thus deficient in pluripotency.

### Telomerase activity and expression of telomerase genes in porcine iPS cells

**Figure 4:**
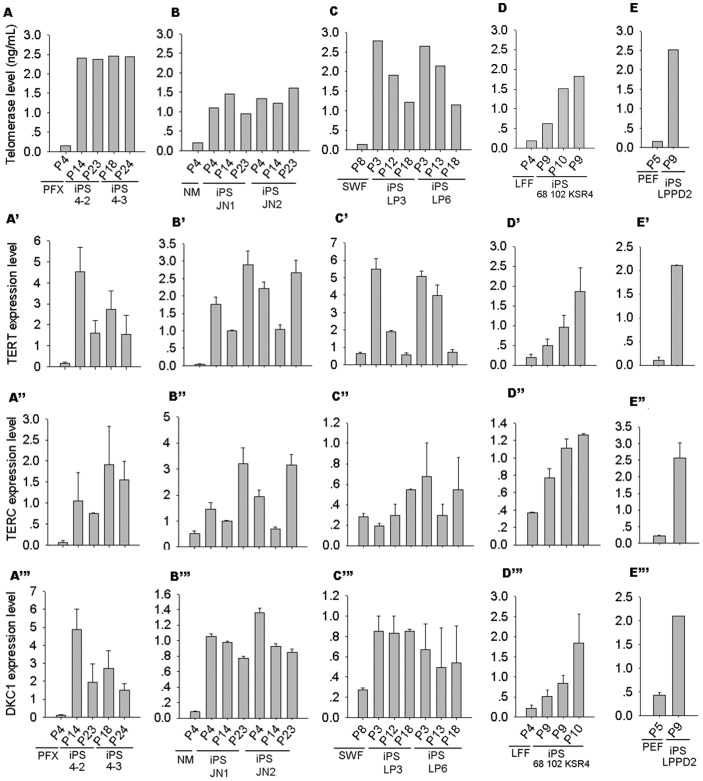
Quantification of telomerase and relative expression levels of telomere genes in porcine iPS cells at various passages, in comparison with their progenitor cells. (A–E) Relative telomerase levels of various iPS cell lines. Relative expression levels of TERT (A’ –E’), TERC (A” –E”) and DKC1 (A’” –E’”) also are compared among different cell lines at various passages with those of progenitor cells. Bars, mean ± S.E. (n = 3 independent replicate).

Telomerase activity increased in porcine iPS cells at early passages (p9-10), but declined after longer passages in most porcine iPS cells. However, iPS4-2 or 4–3 with sustained activation of exogenes maintained higher telomerase activity (Figure 4). Expression of TERT was higher in iPS cells at early passages than in their progenitor cells, but its expression decreased during continued passage (Figure 4A’–C’). TERC and DKC also were expressed at higher levels in porcine iPS cells at early passages, and maintained the high levels during passages (Figure 4A’’–C’’, 4A’’’–C’’’). Levels of TERT were lower in porcine iPS cells with silenced exogenous genes than in those with active exogenous genes at early passages (Figure 4D, 4D’). Likewise, telomerase activity of porcine iPS cells with silenced exogenous genes was not as high as those of iPS cells with active exogenous genes (Figure 4A). Lower expression levels of TERT appeared to coincide with decreased telomerase activity during passage of porcine iPS cells (Figure 4C, 4C’). Reduced telomerase activity and expression levels of TERT corresponded to telomere shortening in iPS LP3 and LP6, in contrast to iPS4-2 and iPS 4–3 that maintained high telomerase activity during passages (Figure 3A and 3C, Figure 4A and 4C). TERT and TERC were also activated in iPS 9–6 and iPS 10–6 and 10–9, but notably reduced in iPS 10–9 at later passage (Figure S3). Telomerase was activated during reprogramming, but it alone seemed not to fully explain telomere variations in porcine iPS cells.

### Distinctive telomere structure in porcine iPS cells

We hypothesized that other mechanisms independent of telomerase also might influence telomere lengths and stability of porcine iPS cells. By telomere FISH analysis, we noticed that frequency of telomere signals –free end indicative of telomere loss was generally low, only slightly increased in iPS cells with inactive exogenes and did not increase in other porcine iPS cells (Figure S4), in contrast to significant telomere loss in porcine somatic cells during passages [45], suggesting that telomere reprogramming occurs and prevents telomere attrition during induction of porcine iPS cells.

**Figure 5:**
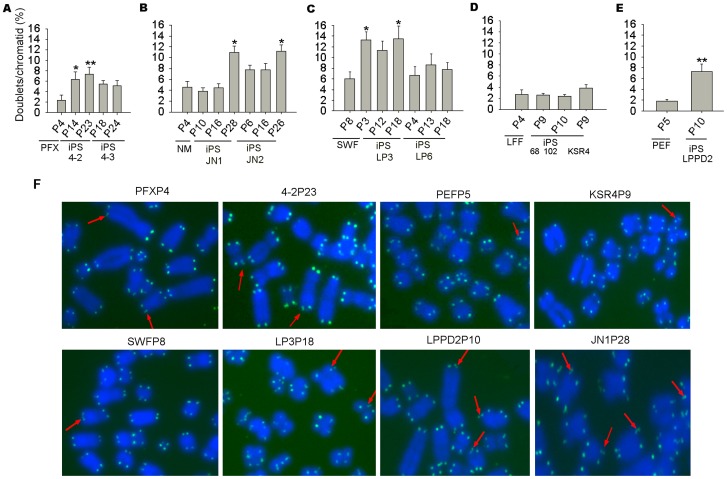
Telomeric doublets following porcine iPS generation. (A–C) Incidence of telomeric doublets increased in iPS cell lines compared with primary cells, PFXP4, NMP4 and SWFP8 respectively. *, p<0.05; **, p<0.01. (D) Frequency of telomere doublets decreased in 68P9, 102P10 and KSR4P9 with exo-genes silencing or partial silencing, compared with progenitor LFFP5. (E) Frequency of telomere doublets increased in LPPD2P10 induced by culture under low oxygen and small molecules, compared with PEF. ** p<0.01. (F) Representative images of telomere FISH and telomere doublets (exampled by red arrows). Blue, chromosomes stained with DAPI; Green, telomeres labeled with PNA probes.

**Figure 6:**
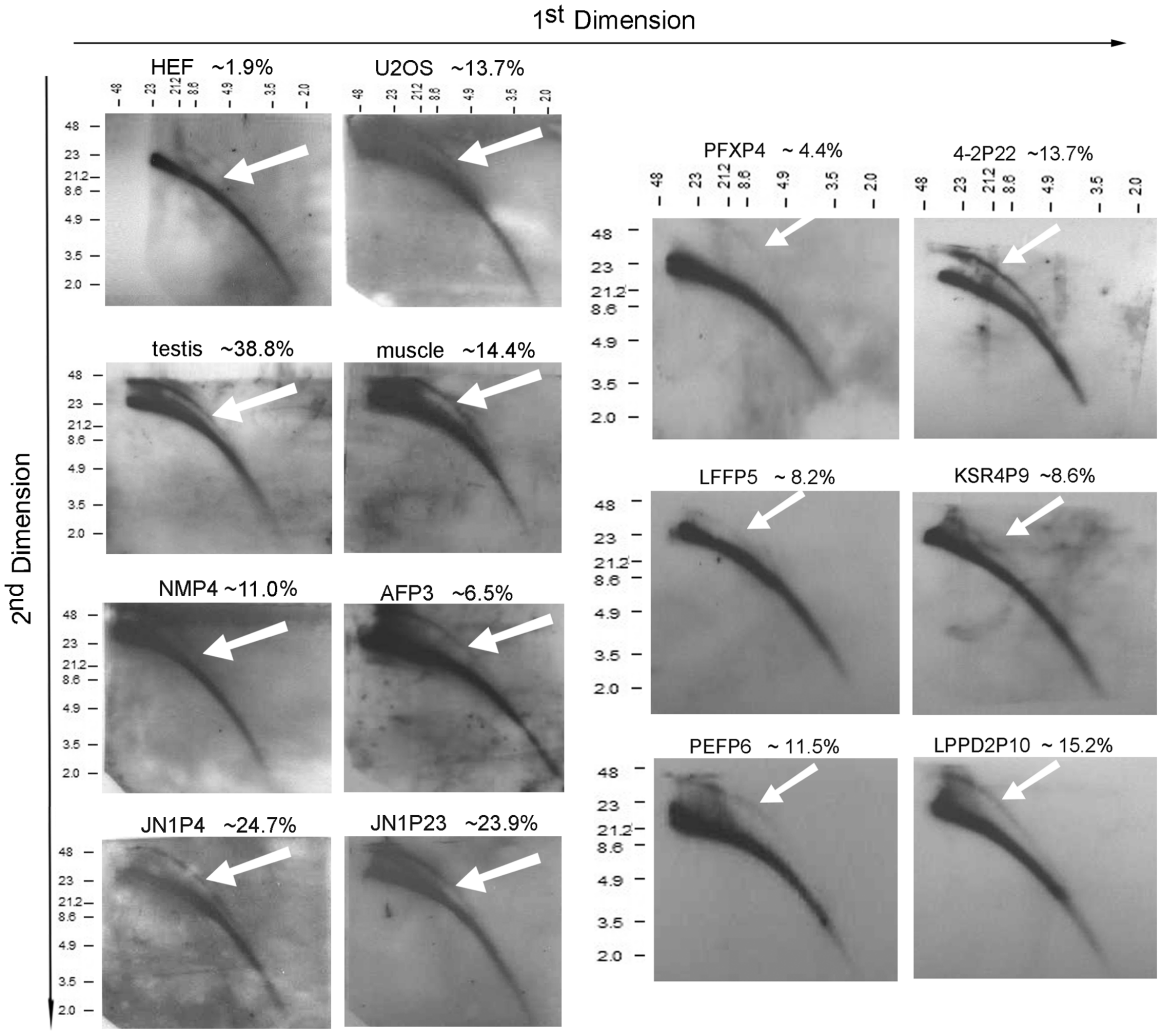
Telomeric circles (t-circles) detected in porcine cells or tissues by neutral-neutral two dimension (2D) gel-electrophoresis. Human embryonic fibroblasts (HEF) and U2OS served as controls. White arrows, t-circles. Percentage of t-circles is shown at the right hand corner on top of each image for relevant cells or tissues. P, passage.

Intriguingly, porcine telomeres showed frequent doublets, which often refer to more than one telomeric signal at single chromatid end [45]–[47]. Porcine iPS cells exhibited increased incidence of telomere doublets compared to their progenitor cells and during passages (Figure 5). Moreover, the occurrence of telomere doublets was higher in porcine iPS cells when the exogenous genes remained active but was almost the same as primary cells in the porcine iPS cells with exogene silencing. Telomere doublets could indicate telomere recombination for telomere maintenance, or telomere damage that eventually results in telomere loss and instability [45], [48], [49], and these features can be assessed by telomere t-circles [50]. 2D gels from porcine primary cells, iPS cells and tissues of testis and muscle were blotted and hybridized with a C-rich telomere probe (see method for details). T-circles were found in both porcine primary cells and iPS cells (Figure 6). To ensure that this result was not caused by *in vitro* culture, genomic DNA from various pig tissues including testis, muscle and spleen were analyzed using the same method and served as controls. Human embryonic fibroblasts and U2OS (ATCC), an alternative lengthening of telomere (ALT) cell line served as negative and positive controls, respectively. As expected, human fibroblasts exhibited very low frequency of t-circles, in contrast to U2OS cells. In contrast, porcine tissues or cells showed higher incidence of t-circles than that of human fibroblasts. Porcine testis displayed even higher occurrence of t-circles than did U2OS cells. Consistent with telomere doublets, telomere t-circles were found at high frequency in porcine cells (Figure 6). Percentage of t-circles was increased even more in porcine cells during passages (Figure 6).

ALT also can be visualized cytogenetically by frequent presence of telomere sister chromatid exchanges (T-SCE) using chromosome orientation fluorescence *in situ* hybridization (CO-FISH) [51]. By CO-FISH, the nascent DNA strand is labeled with BrdU, and then specifically digested by Exonuclease III, revealing a characteristic pattern of terminal sister chromatid staining by telomere specific FISH that can be observed in metaphase spreads [52]. To further investigate putative involvement of recombination-based mechanisms in telomere dynamics in porcine iPS cells, we analyzed T-SCE in a panel of iPS cell lines. Compared to progenitor cells, iPS cells exhibited increased frequencies of T-SCEs (Figure S5). Frequency of T-SCEs declined in LP3 from P5 to P21, coincided with their shortened telomeres during passages, suggesting that T-SCE might be associated with telomere maintenance in porcine iPS cells. iPS cells 68 at P9 and 102 at P10, showed higher frequency of T-SCE than that of their progenitor cells, but their telomeres still were not properly maintained. Thus, T-SCE may occur with telomere reprogramming, but seems to be unable to prevent telomere shortening in porcine iPS cells with insufficient telomerase activation.

### DNA and telomere damage responses in porcine iPS cells

**Figure 7:**
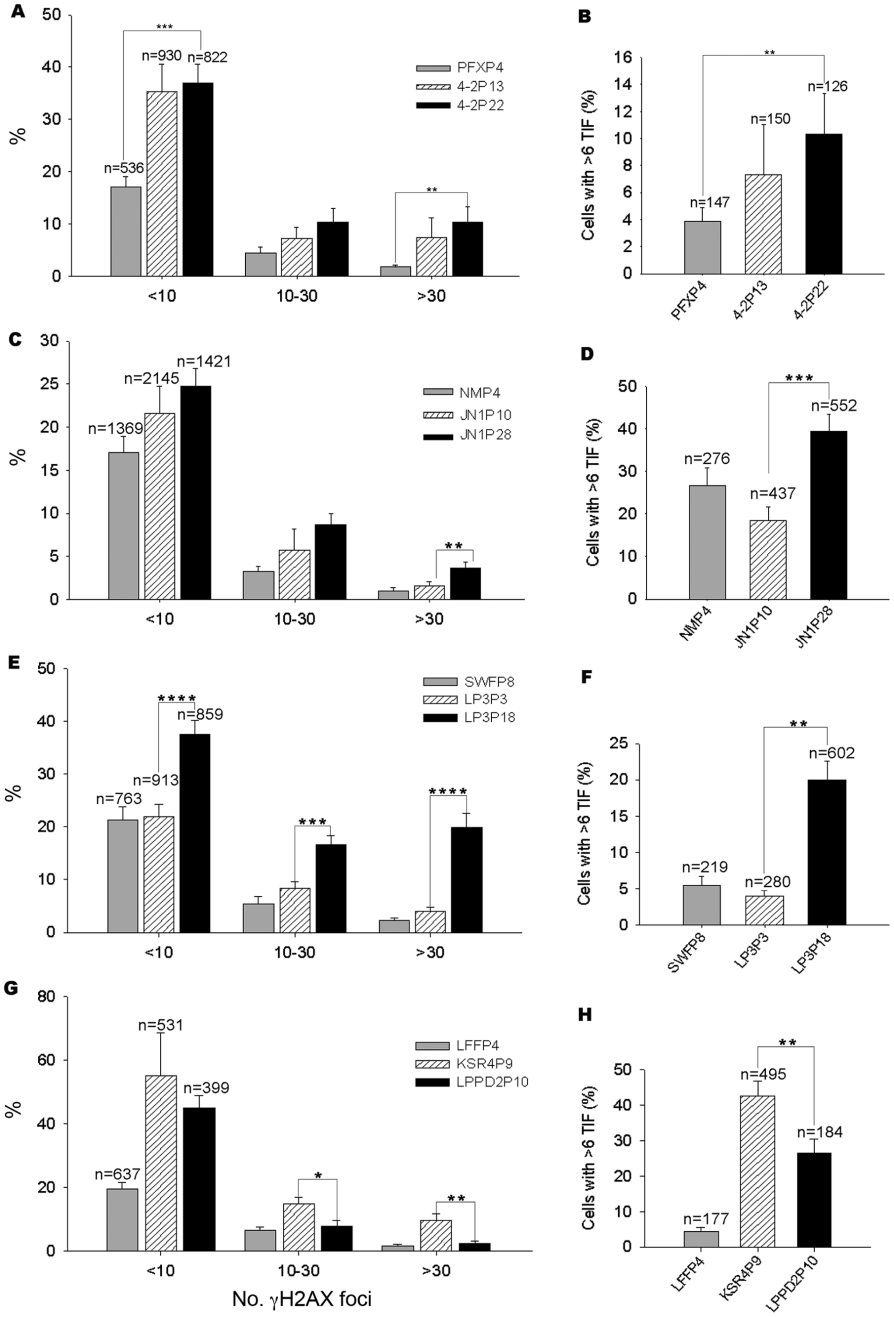
DNA damage and telomere dysfunction-induced foci (TIF) in porcine iPS cells. (A, C, E and G) Percentage of γH2Ax positive cells. Cells are categorized into three groups with fewer than 10, 10–30 and more than 30 γH2Ax foci, respectively. n  =  number of cells counted. *p<0.05, **p<0.01, ***p<0.001, ****p<0.0001 compared with cells at early passage. (B, D, F and H) Percentage of telomere dysfunction-induced foci (TIF). *n*  =  number of cells with γH2Ax positive foci analyzed. **p<0.01, ***p<0.001.

Porcine iPS cells mostly failed to maintain telomeres properly over limited number of passages (about P10). Slow or incomplete telomere reprogramming could initiate terminal DNA damage responses during iPS cell induction, and diminish self-renewal and pluripotency and thus the quality of iPS cells [53], [54]. We assessed telomere dysfunction-induced foci (TIF) at telomeres by γH2AX in various iPS cells and progenitor cells using the method described [54]. The proportion of γH2AX positive cells and TIF foci in various iPS cells increased with passages (Figure 7). Interestingly, the frequency of TIF was lowest in iPS4-2, compared with other iPS cells (Figure 7B, D, F, H). JN1 had relatively high incidence of TIF (Figure 7D). DNA damage shown by γH2AX positive staining and TIF foci both elevated in later passage LP3 P18 compared to earlier passage P3 (Figure 7E, F). iPS KSR4 with silenced exogenes at P5 also showed high frequency of DNA damage and TIFs even at passage P9, compared to the progenitor cells (Figure 7G). iPS cell LPPD2 had reduced DNA and telomere damage response at similar passage (Figure 7H). Coincident with increased frequency of DNA damage and TIFs, chromosomal abnormalities were high in JN1/JN2 iPS cells at later passage (P26-28), and even higher in LP3/LP6 at P18 by karyotype analysis (Table S2). It appears that DNA and telomere damage responses elevate in porcine iPS cells presumably with least reprogramming or maintenance of telomeres.

## Discussion

We show that telomere reprogramming occurs during induction of porcine iPS cells. Porcine iPS cells seem to show mild telomere elongation like human iPS cells [33], [34], unlike remarkable telomere lengthening found in mouse iPS cells [23], [32], [36]. Also, telomere length differs in various porcine iPS cells, consistent with previous reports in mouse and human iPS [32]–[36], [55]. Further, telomeres are elongated and maintained in porcine iPS cell lines with sustained activation of exogenes, in contrast to telomere shortening with passages of iPS cells that have exogene silencing.

Exo-transgene silencing is a prerequisite for normal cell differentiation [56]. Retroviral silencing is important for full reprogramming of somatic cells into iPS cells [57]. Moreover, the timing of exo-transgene silencing correlates with completion of full reprogramming and the quality of iPS cell lines [58]. Interestingly, reactivation of exogenous genes was found in telomerase-deficient mouse iPS cells with telomere dysfunction [36]. Our data suggest that activation of exogenes is associated with insufficient activation of telomerase activity and telomere reprogramming in porcine iPS cells. Silencing or partial silencing of the exogenes can be found in few porcine iPS cells at early passages, yet the exogenes reactivate during passages, coincided with reduced activation of telomerase genes and endogenous pluripotent genes.

Telomerase is activated after porcine iPS generation. Some iPS cells fail to elongate telomeres, in association with inadequate activation of telomerase genes, particularly at early passages, despite their expression of Oct4 and Nanog. When telomerase genes are insufficiently activated, telomeres elongate less even over extended passages. These data are consistent with the notion that activated telomerase is required for telomere maintenance and self-renewal of iPS cells [32], [34], [36]. Telomerase activation during iPS reprogramming is associated with upregulation of TERT, the telomerase RNA component TERC and dyskerin. TERT expression maintains relatively higher levels in iPS cells with stable telomere length during passage, whereas the expression levels of TERT decrease with increasing passages of iPS cells, which also show telomere shortening. Low expression of TERT/telomerase also is associated with telomere shortening in exogene silenced iPS cells.

In addition to telomerase, ALT also plays an important role in telomere length maintenance [59], [60] and may partially compensate for telomere shortening that otherwise occurs due to telomerase insufficiency. Our data suggest that telomerase independent mechanism also may contribute to telomere maintenance of porcine iPS cells, despite to limited extent. One characteristic of ALT-mediated telomere maintenance is the presence of extrachromosomal telomeric repeat-containing DNA circles (t-circles) [50]. Neutral-neutral two-dimensional (2D) gel electrophoresis was used to analyze circular DNA molecules and telomeric repeat-containing DNA circles (t-circles) [61], [62]. Interestingly, t-circles are found in pig genome of various cell types and tissues. T-circles attend ALT of mouse and human [63], by a roll-and spread mechanism [64], and provide specific and quantifiable markers of ALT activity [65]. Yet, t-circles also could be involved in a telomere trimming mechanism that rapidly removes telomere loops and negatively regulates telomere lengths in normal mammalian cells and human cancer cells [50], [61]. T-circles may contribute to maintenance of porcine telomeres at certain length without dramatic telomere elongation, and play dual roles in both lengthening and shortening of telomeres, depending on factors that remain to be identified.

In addition to t-circles, frequency of T-SCEs and telomere doublets also increases in in porcine iPS cells. T-SCEs have been associated with telomere elongation by recombination in mouse ES cells [60]. Reactivation of exogenous transcription factors also coincides with increased rates of T-SCE. Telomeric doublets are more frequent in pig than in mouse and human cells (Figure 5) [45], [47]. Interestingly, the incidence of telomere doublets increases in iPS cells with active exogene expression, and remains during passages. In contrast, the frequency of doublets does not increase in iPS cell lines with exogene silencing at early passages, compared with primary cells (Figure 5D). Exogenous genes may introduce oncogene-induced replication stress that leads to fragile chromosomes and telomeres. Technically, the telomere doublets may interfere with the quantitative FISH for porcine cells, since the average telomere fluorescence signals can be reduced artificially when the two telomere dots completely separate, whereas qPCR amplifies the total telomeres, such that telomere length expressed as T/S ratio shows slight differences (for example, LP3P3 in Figures 2C and 3C), but in general, corresponds to telomere Q-FISH measurements. Increased frequencies of telomere doublets and T-SCE may be associated with telomere maintenance in iPS cells. ALT mechanism could be a response to the insufficient telomerase, but may not sustain telomeres and long-term survival of iPS cells without sufficient telomerase.

Enhanced telomere reprogramming and maintenance remarkably reduces DNA damage responses, increases genomic stability and improves the quality of mouse iPS cells [54]. Indeed, porcine iPS cells iPS4-2 with sustained active exogenes show telomere maintenance and generate cloned piglets by nuclear transfer [21], as well as contribute to fetal chimeras by gestation day 30, consistent with the notion that telomere maintenance is essential for the high quality of iPS cells. Moreover, DNA and telomere damage responses increase with passages of porcine iPS cells, particularly those with reduced expression of pluripotent genes and insufficient activation of telomerase genes. Severe damages to DNA and telomeres may lead to genomic instability as shown by aberrant karyotypes (Table S2), and these also may lead to reduced pluripotency shown by teratoma tests.

Authentic porcine ES cells are still not available as standard references to compare the expression levels of pluripotent genes and telomerase genes; nonetheless, we speculate that active exogenous genes remain to compensate for insufficiently activated endogenous pluripotent genes and telomerase genes, to maintain telomeres and self-renewal of porcine iPS cells. It is also possible that the sub-optimal culture conditions fail to maintain the reprogrammed porcine iPS cells such that exogenes become reactivated again. Further improvement in the induction methods and culture conditions is warranted to fully activate endogenous pluripotent genes and to maintain telomeres for achieving authentic pluripotent porcine iPS cells.

## Materials and Methods

### Porcine iPS cells

Primary porcine fetal or newborn fibroblasts and newborn bone marrow cells were isolated and cultured as described [45], [47]. Primary cells were transfected by pMXs-based retroviral vectors or Dox-inducible, lentivirus vectors containing mouse, human, monkey or porcine factors (Oct4, Sox2, Klf4, c-Myc) (detailed in Table 1). Porcine iPS cell lines 4–2 and 4–3 were generated using Dox-inducible, lentivirus human factors and cultured in human ES culture condition [12], [21]. iPS LP3 and LP6 were generated by retroviral transduction of human factors [15]. iPS 68 and 102 were generated from monkey factors and cultured in human ES medium [66], [67]. iPS JN1 and JN2 were generated using mouse factors, and KSR4 and LPPD2 from porcine four factors [21], [47]. LPPD2 cell lines were cultured with 5% O_2_. For alkaline phosphatase (AP) assay, 10,000 cells were plated in a 6-well plate, and the formed colonies assessed using the Vector blue kit from Vector Laboratories (DAKO, Carpinteria, CA).

### Immunofluorescence microscopy

iPS cells were washed twice in phosphate buffered saline (PBS), then fixed in freshly prepared 3.7% paraformaldehyde in PBS (pH 7.4), permeabilized in 0.1% Triton X-100 in blocking solution (3% goat serum in PBS) for 30 min, washed three times, and left in blocking solution for 1 h. iPS cells were incubated overnight at 4°C with primary antibodies against Oct4 (sc5279, Santa Cruz, CA), Nanog (Abcam, ab10626), SSEA-4, TRA-1-60 and TRA-1-81 followed by incubation for 1 h with secondary antibodies, Alexa fluor 568 goat anti-rabbit IgG (Molecular Probes, Invitrogen) or Alexa fluor 488 goat anti-mouse IgG or IgM (Molecular Probes, Invitrogen) diluted in 1:200 with blocking solution. Samples were washed and counterstained with 0.5 µg/ml Hoechst 33342 in Vectashield mounting medium. Fluorescence was detected and imaged using a Zeiss motorized Axio Imager Z1 fluorescence microscope.

### Teratoma formation test

One million iPS cells were injected subcutaneously into each flank of recipient NOD/SCID mice (Jackson Laboratory, Bar Harbor, ME, http://www.jax.org). Paraffin sections of formalin-fixed teratoma specimens were prepared 4–8 weeks after injection, and analysis of hematoxylin & eosin-stained tissue sections was performed for each specimen. All animal experiments were performed in accordance with the guidelines for use of the animals for this research approved by the Nankai Institutional Animal Care and Use Committee.

### Telomere measurement by quantitative real-time PCR

The average telomere length was measured from total genomic DNA by real-time PCR assay, based on the method described [23], [68], [69], with slight modifications for measurement of pig telomeres [45]. All PCR reactions were performed on the iCycler iQ real-time PCR detection system (Bio-Rad, Hercules, CA, USA) using telomeric primers (5′–3′): forward, CGGTTTGTTTGGGTTTGGGTTTGGGTTTGGGTTTGGGTT; reverse, GGCTTGCCTTACCCTTACCCTTACCCTTACCCTTACCCT. Primers for the reference control gene (pig 36B4 single-copy gene): forward, TGAAGTGCTTGACATCACCGAGGA; reverse, CTGCAGACATACGCTGGCAACATT. Both telomeres and the 36B4 gene were amplified under the same conditions. For each PCR reaction, a standard curve was made by serial dilution of known amounts of DNA. The telomere (T) signal was normalized to the signal from the single-copy (S) gene to generate a T/S ratio indicative of the relative telomere length. Equal amounts of DNA (35 ng) were used for each reaction, with at least 3 replicates for each specimen.

### Telomere quantitative fluorescence *in situ* hybridization (Q-FISH)

Telomere FISH and quantification were performed as described previously [70], [71], except for a fluorescein isothiocyanate (FITC)-labeled (CCCTAA) peptide nucleic acid (PNA) probe used in this study [45]. Cells were incubated with 0.2–0.3 μg/mL nocodazole (Sigma, St. Louis, MO, USA) for 3 h to enrich the cells in metaphase for making chromosome spreads. Metaphase-enriched cells were subjected to hypotonic treatment in a 75 mM KCl solution, fixed with methanol:glacial acetic acid (3:1), and spread onto clean slides. Telomeres were denatured at 80°C for 3 min and hybridized with telomere PNA probe (0.5 μg/mL) (Panagene, Daejeon, Korea). Chromosomes were stained with 0.5 μg/mL 4′,6-diamidino-2-phenylindole (DAPI). Fluorescence signals were digitally imaged using a Zeiss microscope with FITC/DAPI filter sets, in combination with AxioCam and AxioVision software 4.6. For quantitative measurement of telomere length, telomere fluorescence intensity was integrated using the TFL-TELO program (a gift kindly provided by P. Lansdorp, Terry Fox Laboratory, Vancouver, Canada). More than 10 metaphase spreads were examined from each cell line.

### Telomere dysfunction-induced foci (TIFs) by immunofluorescence (IF)-telomere FISH (IF-FISH)

IF-FISH was performed as described previously [45], [48]. Briefly, cells were grown on gelatin-treated cover slips and fixed with 2% paraformaldehyde for 10 min at room temperature. The cells were washed with a blocking solution (1 mg/mL bovine serum albumin, 3% goat serum, 0.1% Triton X 100 and 1 mM EDTA pH 8.0) and incubated with anti-γH2AX (Upstate, 05–636, CA) in blocking solution. The secondary antibody against mouse IgG was labeled with Alexa Flour 594 (Invitrogen). Cells were fixed again in 2% paraformaldehyde for 5 min, and FISH performed using a FITC-(CCCTAA)_3_ PNA telomere probe (Panagene), as described above. DNA was counterstained with 0.5 μg/L Hoechst 33342 in Vectashield mounting medium (Vector Laboratories, Burlingame, CA). Fluorescence was detected and imaged using a Zeiss Imager Z1 microscope equipped with an epifluorescence source and lenses.

### Chromatid Orientation Fluorescence *In Situ* Hybridization (CO-FISH)

Analysis of telomeric Sister Chromatid Exchanges (T-SCEs) was performed according to Bailey et al. [51], with minor modifications. Metaphase spreads were prepared by as above. Chromosome preparations were treated with 0.5 mg/ml RNase-A (Roche) for 10 min at 37°C, stained with Hoechst 33258 (0.5 μg/ml) (Sigma), incubated in 2xSSC (Invitrogen) for 15 min at room temperature (RT) and exposed to 365-nm UV light (Stratalinker 1800 UV irradiator) for 30 min. The 5′-bromo-2′-deoxyuridine-substituted DNA was digested with Exonuclease III (Promega) in a buffer supplied by the manufacturer (5 mM DTT, 5 mM MgCl2 and 50 mM Tris-HCl, pH 8.0) for 10 min at RT. The slides were then dehydrated through a cold ethanol series (70%, 85% and 100%) and air-dried. PNA-telomere strand specific FISH was performed using heat denaturated Fluorescein-OO-(CCCTAA)3 and TAMRA (6-carboxytetramethylrhodamine)-OO-(TTAGGG)3 (Bio-Synthesis Inc, TX, USA) telomeric probes. After hybridization and washes, chromosomes were stained with DAPI and imaged using a Zeiss Axio-Imager Z1 microscope and the Metasystems Isis Software (Zeiss, Germany).

### Gene expression by real-time PCR

Total RNA was isolated from fibroblasts, mesenchymal cells and iPS cells using RNeasy mini kit (Qiagen). 2 µg of RNA were subjected to cDNA synthesis using M-MLV Reverse Transcriptase (Invitrogen). Real-time quantitative PCR reactions were set up in duplicate with the SYBR Green Master Mix (TOYOBO) and run on the iCycler iQ5 2.0 Standard Edition Optical System (Bio-Rad). Each sample was repeated 3 times and analyzed with β-actin as the internal control. Primers were designed using IDT DNA website (http://www.idtdna.com/Home/Home.aspx) or Oligo6 software. Telomerase related gene were amplified with primers, 5′ –GAAAGCCAGAAACGCAGGGAT-3′, and 5′- CCCAGAAGACAGCTGTAGGTAACG-3′ for TERT; 5′ –GTCTAACCCTAACTGAAAGAGGCG-3′, and 5′- CCCAGAAGACAGCTGTAGGTAACG-3′ for TERC; 5′ –ACATGGTGACGATGCATGATGTGC -3′, and 5′- ATGGCATTGACCGCACTGTCTTTC -3′ for DKC1; 5′ –TGCGGCATCCACGAAACTAC -3′, and 5′- TTCTGCATCCTGTCGGCGAT -3′ for actin. Primers for quantitative PCR analysis of total– or endo- and exo-gene expression are provided in Table S1. Experiments were independently repeated three times.

### Neutral-neutral two-dimensional gel electrophoresis

Genomic DNA extracted form pig tissues and cells was frozen at – 20 °C until use. Neutral-neutral two-dimensional gel electrophoresis was performed based on the protocols established by Brewer and Fangman (http://fangman-brewer.genetics.washington.edu/2Dgel.html), with minor modifications. Nine micrograms of genomic DNA was separated on a 0.4% low-EEO agarose gel in 1× Tris-borate- EDTA at 12 V/cm for 18 h at room temperature. Lanes containing samples were cut and placed to the direction of electrophoresis, and 1.0% agarose gel was poured on the first lane. The second dimension was run at 60 V/cm for 4.5 h at room temperature. The DNA was transferred to the positive Nylon membrane (Amersham) and hybridized with c-rich telomere probes with DIG (Roche). After washing, the membrane was incubated with Anti-DIG-AP working solution. Images were captured on a Medical X-ray Processor by exposure for 5–10 min. Quantification of t-circles was performed as described [72].

### Statistical Analysis

Percentages were transformed using arcsine transformation. Transformed percentage data and other numbers were analyzed by analysis of variance (ANOVA), and means were compared by Fisher's protected least-significant difference (PLSD) test using StatView software from SAS Institute Inc. (Cary, NC). Significant differences were defined as p<0.05, 0.01, or lower.

## Supporting Information

Figure S1 **Expression of endogenous and exogenous genes of iPSCs determined by real-time PCR analysis.** (A) Expression of endogenous genes (endo-) Oct4, Sox2, Klf4, v-Myc and Lin28 in iPS JN1 and JN2 cell lines compared with primary cells NMP4. P, passage. (B) Expression of exogenous genes (exo-) Oct4, Sox2, Klf4 c-Myc and Lin28 in iPS JN1 and JN2 cell lines compared with NMP4 served as negative control. NMP4 at day 5 following transfection of the four Yamanaka factors served as positive control. (C) Expression of endogenous (endo-) Oct4, Sox2, Klf4, c-Myc and Lin28 in iPS cells LPPD2 at P10, then cultured with small molecules for 5 passages, compared with iPS in normal culture condition. (D) Expression of exogenous genes (exo-) Oct4, Sox2, Klf4, and c-Myc in iPS cells cultured with small molecules compared with iPS in normal culture condition. Bars, mean ± SE.
(DOC)

Figure S2 **Expression of pluripotent genes Oct4, Sox2, Klf4, vMyc, and Nanog in porcine iPS cell lines 4–2 (A), LP3 (B), KSR4 (C) and LPPD2 (D) by quantitative real-time PCR.** *p<0.05. **p<0.001 compared with LP3P3 (B). LFFP5 and PEFP5 used in (C) and (D), respectively are progenitor fibroblasts at day5 after transfection. to, total levels; ex, expression levels of exogenous genes Oct4, Sox2, Klf4, and vMyc. P, passage. Bars, mean ± SE (n = 3 independent replicate).
(DOC)

Figure S3 **Relative expression levels of telomerase-associated genes TERT, TERC and DKC1 in porcine iPS cell lines 9–6, 10–6, 10–9 during passages, in comparison with their progenitor cells PEFL (porcine embryonic fibroblast isolated from Nong Da Xiang mini-pig).** Bars, mean ± S.E. (n = 3 independent replicate).
(DOC)

Figure S4 **Frequency of telomere signal-free ends/chromatid, indicative of telomere loss in various porcine cell types.** (A) Representative image of Q-FISH showing signal-free ends. Blue, chromosomes stained with DAPI; Green, telomeres labeled with PNA probes. White arrows, signal-free ends. (B–G) Percentage of telomere signal-free ends in different cell lines.
(DOC)

Figure S5 **Telomere sister chromatid exchange (T-SCE) of different iPS cells detected by chromosome orientation fluorescence** ***in situ*** **hybridization (CO-FISH**)**.** (A) Representative image of CO-FISH. Blue, DAPI-stained chromosomes. Green dots, C-rich telomeric sequences; red dots, G-rich telomeric sequences. White arrowheads, T-SCEs. (B) Frequency of T-SCEs increased after iPS generation.
(DOC)

Table S1 **Primers for endo- and exo- gene expression by quantitative real-time PCR.**
(DOC)

Table S2 **Karyotypes of porcine primary cells and iPS cells at various passages.**
(DOC)
