# Correction: Telomere Reprogramming and Maintenance in Porcine iPS Cells

**DOI:** 10.1371/annotation/f5e4554b-18cc-46ef-ac39-73ac4d6750ae

**Published:** 2013-11-14

**Authors:** Guangzhen Ji, Weimin Ruan, Kai Liu, Fang Wang, Despoina Sakellariou, Jijun Chen, Yang Yang, Maja Okuka, Jianyong Han, Zhonghua Liu, Liangxue Lai, Sarantis Gagos, Lei Xiao, Hongkui Deng, Ning Li, Lin Liu

Figure 1H was labeled incorrectly. iPS JN1 should be iPS LPPD2. Please see the corrected Figure 1 here: 

**Figure:**
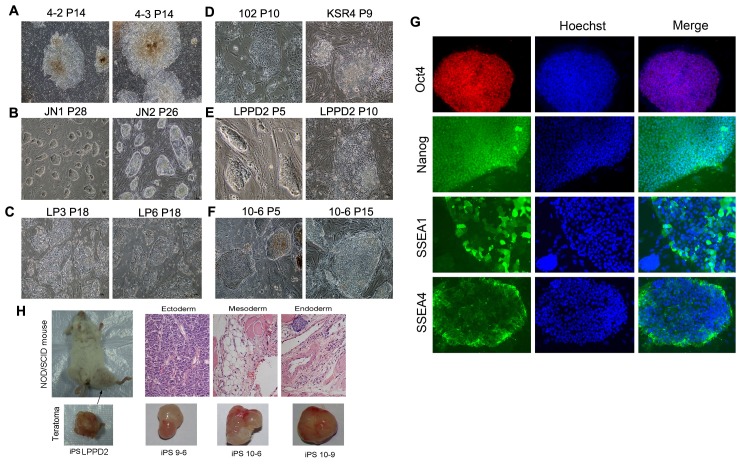


The corrected text for (H) in the Figure 1 legend is: (H) Teratomas formed from pig iPS cells. Shown are three typical germ layers by histological section of teratomas from iPS LPPD2, and teratomas from iPS 9 and iPS 10 (weight around 0.5–1.4 g).

The reference to Figure 1H in the Results section is also affected. In the first paragraph "Porcine iPS cells JN1 at early passages could form teratomas and differentiate into cell types of three germ layers in vitro (Figure 1H, Table 1)" should read: "Porcine iPS cells LPPD2 at early passages could form teratomas and differentiate into cell types of three germ layers (Figure 1H, Table 1)."

