# Supplementary material for: Telomere Reprogramming and Maintenance in Porcine iPS Cells
Source: PLoS One. 2013 Sep 30;8(9):e74202. doi: 10.1371/journal.pone.0074202 (PMC3787036; doi:10.1371/journal.pone.0074202)
Supplement: Figure S1 — Expression of endogenous and exogenous genes of iPSCs determined by real-time PCR analysis. (A) Expression of endogenous genes (endo-) Oct4, Sox2, Klf4, v-Myc and Lin28 in iPS JN1 and JN2 cell lines compared with primary cells NMP4. P, passage. (B) Expression of exogenous genes (exo-) Oct4, Sox2, Klf4 c-Myc and Lin28 in iPS JN1 and JN2 cell lines compared with NMP4 served as negative control. NMP4 at day 5 following transfection of the four Yamanaka factors served as positive control. (C) Expression of endogenous (endo-) Oct4, Sox2, Klf4, c-Myc and Lin28 in iPS cells LPPD2 at P10, then cultured with small molecules for 5 passages, compared with iPS in normal culture condition. (D) Expression of exogenous genes (exo-) Oct4, Sox2, Klf4, and c-Myc in iPS cells cultured with small molecules compared with iPS in normal culture condition. Bars, mean ± SE. (DOC) [file pone.0074202.s001.doc]

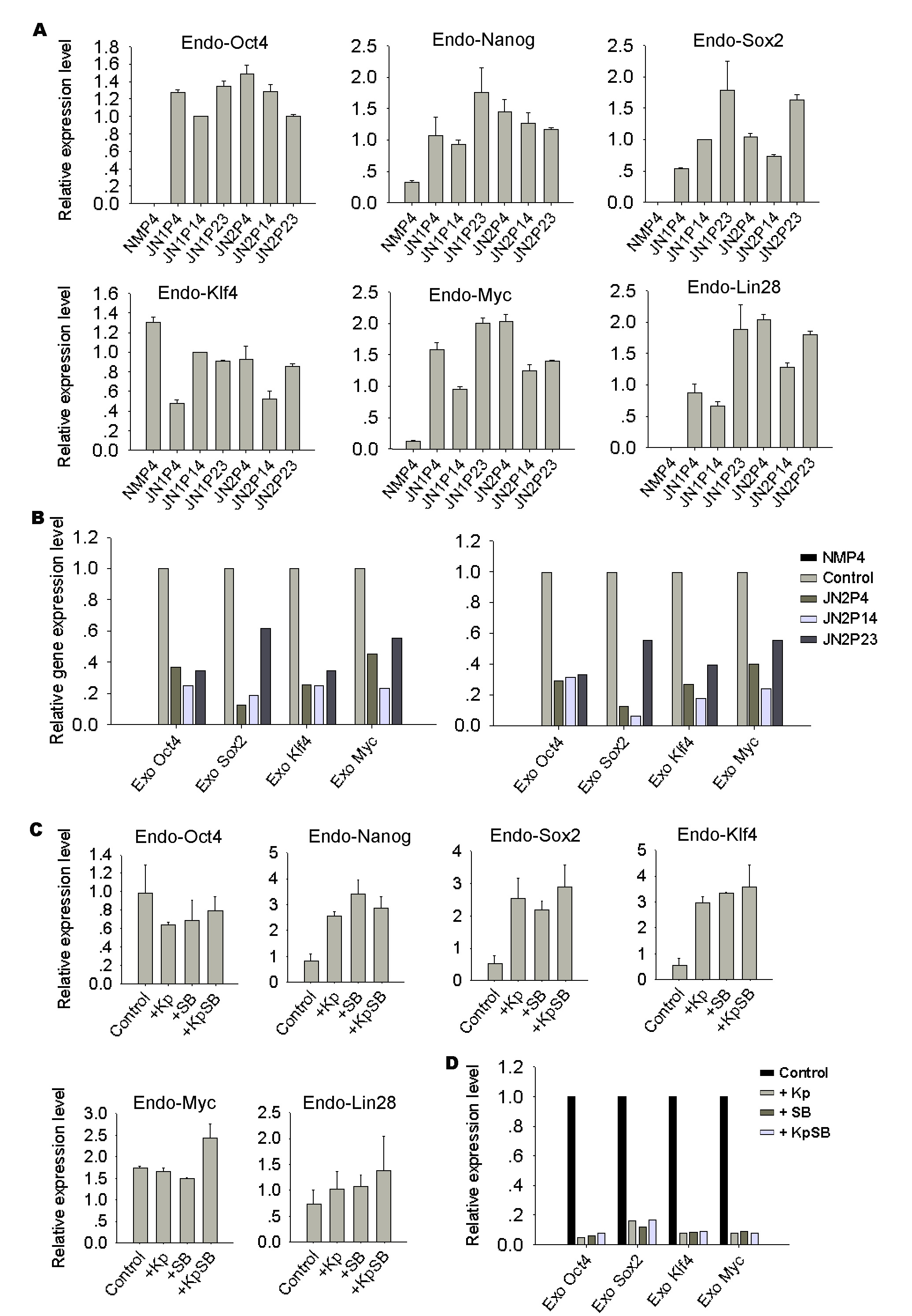


**Figure S1.** Expression of endogenous and exogenous genes of iPSCs determined by real-time PCR analysis. (A) Expression of endogenous genes (endo-) Oct4, Sox2, Klf4, v-Myc and Lin28 in iPS JN1 and JN2 cell lines compared with primary cells NMP4. P, passage. (B) Expression of exogenous genes (exo-) Oct4, Sox2, Klf4 c-Myc and Lin28 in iPS JN1 and JN2 cell lines compared with NMP4 served as negative control. NMP4 at day 5 following transfection of the four Yamanaka factors served as positive control. (C) Expression of endogenous (endo-) Oct4, Sox2, Klf4, c-Myc and Lin28 in iPS cells LPPD2 at P10, then cultured with small molecules for 5 passages, compared with iPS in normal culture condition. (D) Expression of exogenous genes (exo-) Oct4, Sox2, Klf4, and c-Myc in iPS cells cultured with small molecules compared with iPS in normal culture condition. Bars, mean ± SE.
