# Supplementary material for: Telomere Reprogramming and Maintenance in Porcine iPS Cells
Source: PLoS One. 2013 Sep 30;8(9):e74202. doi: 10.1371/journal.pone.0074202 (PMC3787036; doi:10.1371/journal.pone.0074202)
Supplement: Figure S2 — Expression of pluripotent genes Oct4, Sox2, Klf4, vMyc, and Nanog in porcine iPS cell lines 4–2 (A), LP3 (B), KSR4 (C) and LPPD2 (D) by quantitative real-time PCR. *p<0.05. **p<0.001 compared with LP3P3 (B). LFFP5 and PEFP5 used in (C) and (D), respectively are progenitor fibroblasts at day5 after transfection. to, total levels; ex, expression levels of exogenous genes Oct4, Sox2, Klf4, and vMyc. P, passage. Bars, mean ± SE (n = 3 independent replicate). (DOC) [file pone.0074202.s002.doc]

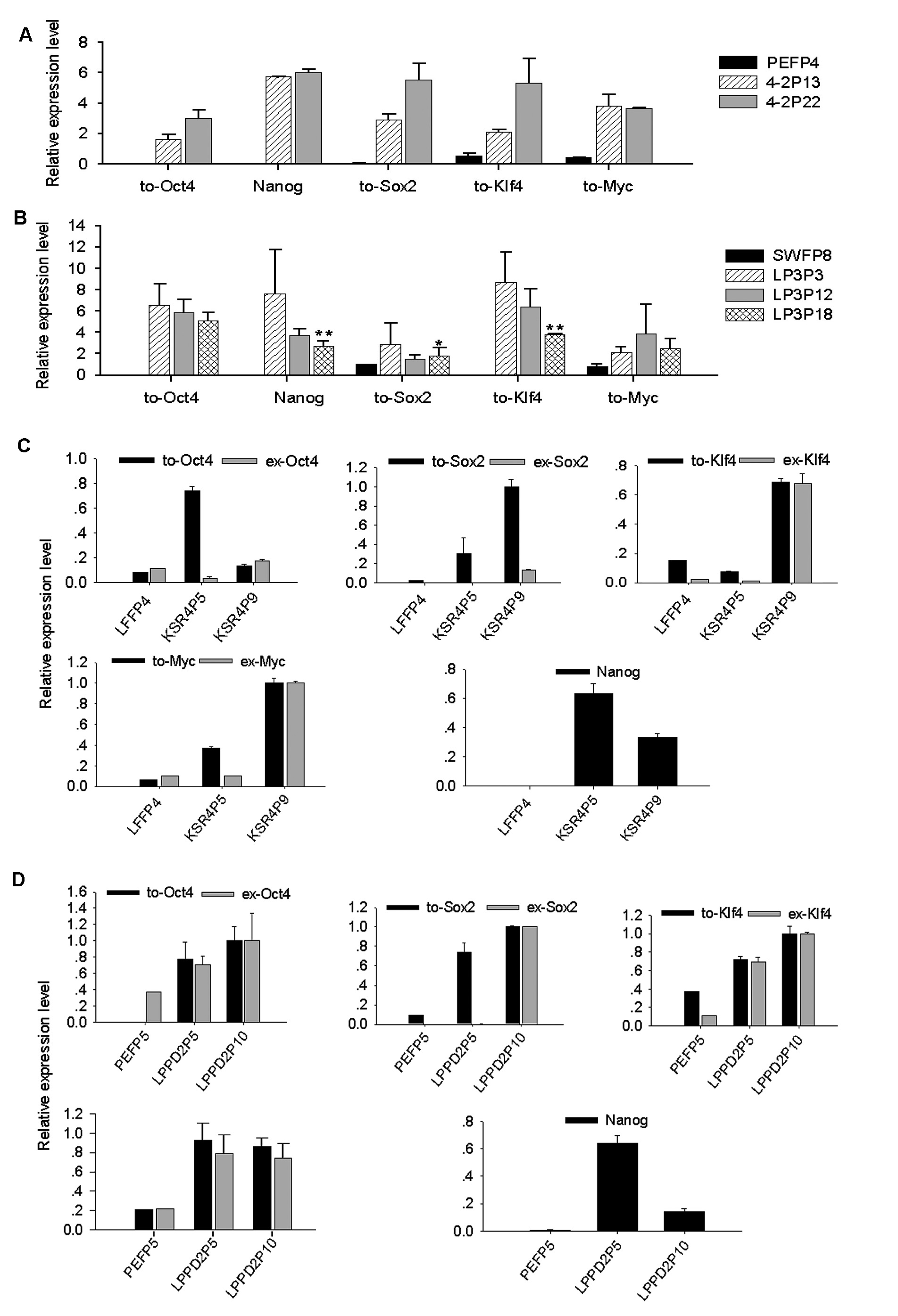
**Figure S2.** Expression of pluripotent genes Oct4, Sox2, Klf4, vMyc, and Nanog in porcine iPS cell lines 4-2 (A), LP3 (B), KSR4 (C) and LPPD2 (D) by quantitative real-time PCR. *p<0.05. **p<0.001 compared with LP3P3 (B). LFFP5 and PEFP5 used in (C) and (D), respectively are progenitor fibroblasts at day5 after transfection. to, total levels; ex, expression levels of exogenous genes Oct4, Sox2, Klf4, and vMyc. P, passage. Bars, mean ± SE (n=3 independent replicate).
