# Supplementary material for: Telomere Reprogramming and Maintenance in Porcine iPS Cells
Source: PLoS One. 2013 Sep 30;8(9):e74202. doi: 10.1371/journal.pone.0074202 (PMC3787036; doi:10.1371/journal.pone.0074202)
Supplement: Figure S3 — Relative expression levels of telomerase-associated genes TERT, TERC and DKC1 in porcine iPS cell lines 9–6, 10–6, 10–9 during passages, in comparison with their progenitor cells PEFL (porcine embryonic fibroblast isolated from Nong Da Xiang mini-pig). Bars, mean ± S.E. (n = 3 independent replicate). (DOC) [file pone.0074202.s003.doc]

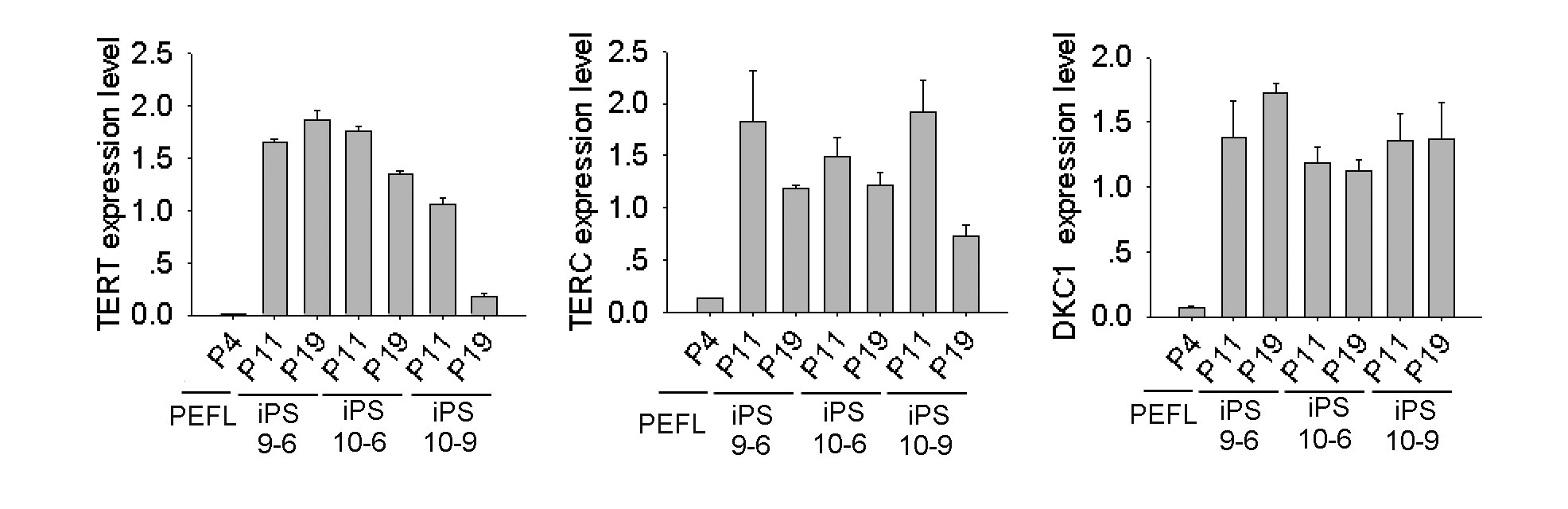


**Figure S3.** Relative expression levels of telomerase-associated genes TERT, TERC and DKC in porcine iPS cell lines 9-6, 10-6, 10-9 during passages, in comparison with their progenitor cells PEFL (porcine embryonic fibroblast isolated from Nong Da Xiang mini-pig). Bars, mean ± S.E. (n=3 independent replicate).
