# Supplementary material for: Telomere Reprogramming and Maintenance in Porcine iPS Cells
Source: PLoS One. 2013 Sep 30;8(9):e74202. doi: 10.1371/journal.pone.0074202 (PMC3787036; doi:10.1371/journal.pone.0074202)
Supplement: Figure S4 — Frequency of telomere signal-free ends/chromatid, indicative of telomere loss in various porcine cell types. (A) Representative image of Q-FISH showing signal-free ends. Blue, chromosomes stained with DAPI; Green, telomeres labeled with PNA probes. White arrows, signal-free ends. (B–G) Percentage of telomere signal-free ends in different cell lines. (DOC) [file pone.0074202.s004.doc]

**
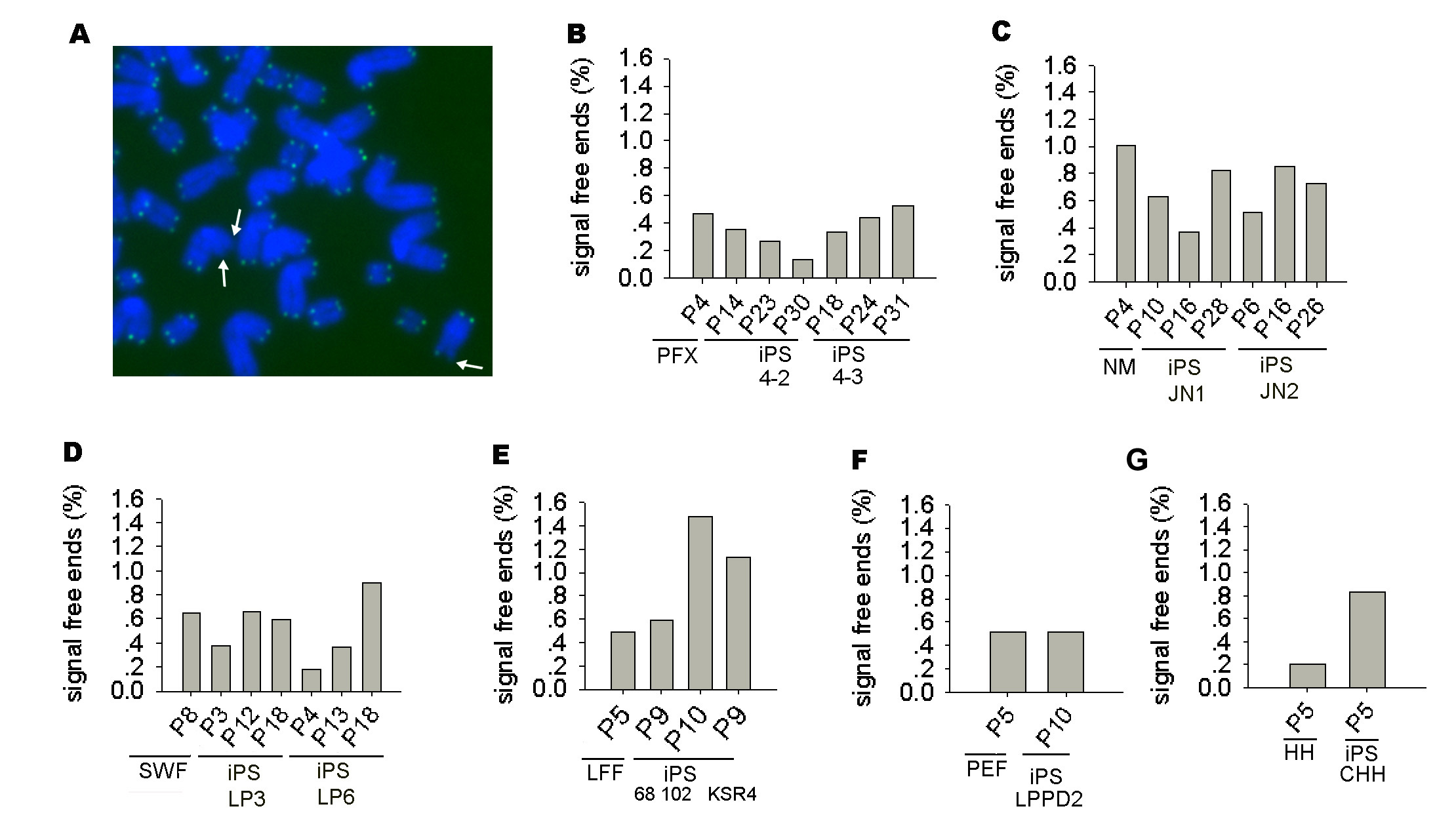
**

**Figure S4.** Frequency of telomere signal-free ends/chromatid, indicative of telomere loss in various porcine cell types. (A) Representative image of Q-FISH showing signal-free ends. Blue, chromosomes stained with DAPI; Green, telomeres labeled with PNA probes. White arrows, signal-free ends. (B-G) Percentage of telomere signal-free ends in different cell lines.
