# Supplementary material for: Telomere Reprogramming and Maintenance in Porcine iPS Cells
Source: PLoS One. 2013 Sep 30;8(9):e74202. doi: 10.1371/journal.pone.0074202 (PMC3787036; doi:10.1371/journal.pone.0074202)
Supplement: Figure S5 — Telomere sister chromatid exchange (T-SCE) of different iPS cells detected by chromosome orientation fluorescence in situ hybridization (CO-FISH). (A) Representative image of CO-FISH. Blue, DAPI-stained chromosomes. Green dots, C-rich telomeric sequences; red dots, G-rich telomeric sequences. White arrowheads, T-SCEs. (B) Frequency of T-SCEs increased after iPS generation. (DOC) [file pone.0074202.s005.doc]

**
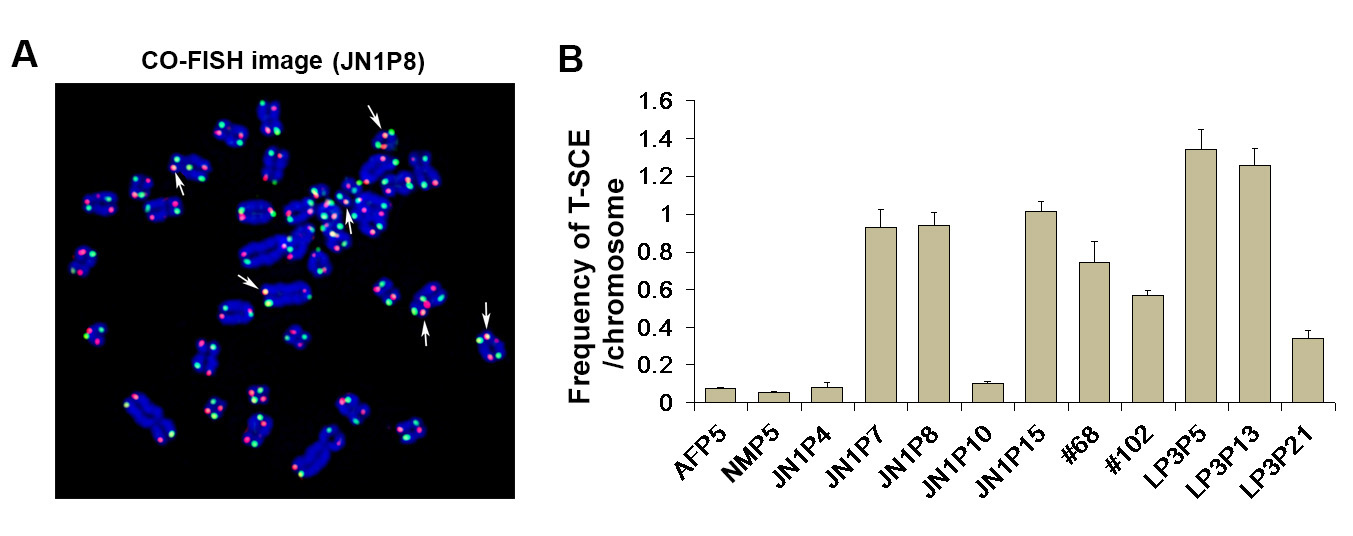
**

**Figure S5.** Telomere sister chromatid exchange (T-SCE) of different iPS cells detected by chromosome orientation fluorescence *in situ* hybridization (CO-FISH). (A) Representative image of CO-FISH. Blue, DAPI-stained chromosomes. Green dots, C-rich telomeric sequences; red dots, G-rich telomeric sequences. White arrowheads, T-SCEs. (B) Frequency of T-SCEs increased after iPS generation.
