# Supplementary material for: Telomere Reprogramming and Maintenance in Porcine iPS Cells
Source: PLoS One. 2013 Sep 30;8(9):e74202. doi: 10.1371/journal.pone.0074202 (PMC3787036; doi:10.1371/journal.pone.0074202)
Supplement: Table S1 — Primers for endo- and exo- gene expression by quantitative real-time PCR. (DOC) [file pone.0074202.s006.doc]

**Table S1.** Primers for endo- and exo- gene expression by quantitative real-time PCR.

| Gene | Primers | |
| --- | --- | --- |
|  | Forward (5’-3’) | Reverse (5’-3’) |
| en-Oct4 | AGGTGTTCAGCCAAACGACCATCT | TCTCGTTGTTGTCAGCTTCCTCCA |
| Nanog | AGGACAGCCCTGATTCTTCCACAA | TCTTCTGCTTCTTGACTGGGACCT |
| en-Sox2 | AGAAGAACAGCCCAGACCGAGTTA | GCTGATCTCCGAGTTGTGCATCTT |
| en-Klf4 | ACCTGGCAGACATCAACGATGTGA | TCAACACAAACTTGCCCATCAGCC |
| en-Myc | CCAGCGAGGATATCTGGAAG | CTCGGTCACCATCTCCAACT |
| ex-mOct4 | CCCAGTGTGGTGGTACGGGAAATC | AGTTGCTTTCCACTCGTGCT |
| ex-mSox2 | CCCAGTGTGGTGGTACGGGAAATC | TCTCGGTCTCGGACAAAAGT |
| ex-mMyc | CCCAGTGTGGTGGTACGGGAAATC | GCTCGCTCTGCTGTTGCTGGTGATAG |
| ex-mKlf4 | CCCAGTGTGGTGGTACGGGAAATC | GTCGTTGAACTCCTCGGTCT |
| PMXs1811-F | GACGGCATCGCAGCAGCTTGGATACAC | |
| ex-pOct4-R | GAGAAGGCGAAGTCGGAAG | |
| ex-pSox2-R | GGCTGTTCTTCTGGTTGC | |
| ex-pMyc-R | GTCTTTGCTTCATGTGGG | |
| ex-pKlf4-R | GTTGGTGAAGCTGACGTTG | |
