# Supplementary material for: Telomere Reprogramming and Maintenance in Porcine iPS Cells
Source: PLoS One. 2013 Sep 30;8(9):e74202. doi: 10.1371/journal.pone.0074202 (PMC3787036; doi:10.1371/journal.pone.0074202)
Supplement: Table S2 — Karyotypes of porcine primary cells and iPS cells at various passages. (DOC) [file pone.0074202.s007.doc]

**Table S2.** Karyotypes of porcine primary cells and iPS cells at various passages.

| Primary Cell or iPS lines | Passage | No. with 38 chromosomes | No. with abnormal chromosomes | Normal (%) |
| --- | --- | --- | --- | --- |
| PFX | P4 | 18 | 4 | 82 |
| iPS  4-2 | P13 | 15 | 2 | 88 |
|  | P22 | 16 | 4 | 80 |
| iPS  4-3 | P17 | 17 | 4 | 81 |
|  | P23 | 18 | 3 | 86 |
| NM | P4 | 15 | 4 | 79 |
| iPS JN1 | P10 | 12 | 2 | 86 |
|  | P16 | 16 | 4 | 80 |
|  | P28 | 11 | 5 | 69 * |
| iPS JN2 | P6 | 17 | 4 | 81 |
|  | P16 | 13 | 3 | 81 |
|  | P26 | 11 | 8 | 57 * |
| SWF | P8 | 10 | 2 | 83 |
| iPS LP3 | P3 | 21 | 7 | 75 |
|  | P12 | 13 | 6 | 68 |
|  | P18 | 5 | 21 | 19 * |
| iPS LP6 | P4 | 16 | 7 | 70 |
|  | P3 | 14 | 8 | 67 |
|  | P18 | 7 | 16 | 34 * |
| LFF | P4 | 11 | 3 | 79 |
| iPS KSR4 | P5 | 10 | 1 | 91 |
|  | P9 | 14 | 4 | 78 |
| iPS 68 | P9 | 17 | 7 | 71 |
| iPS 102 | P10 | 16 | 4 | 80 |
| PEF | P5 | 16 | 5 | 76 |
| iPS LPPD2 | P5 | 15 | 7 | 68 |
|  | P10 | 14 | 3 | 82 |
| HH | P5 | 17 | 6 | 74 |
| iPS CHH | P5 | 10 | 5 | 67 |

* denotes increased frequency of chromosome abnormality with increasing passages.

Primary porcine cells: PFX, new born pig ear fibroblast; NM, mesenchymal cells form new born pig bone marrow; SWF, embryonic pig fibroblast; LFF, embryonic pig fibroblast of Taihu breed; PEF, pig embryonic fibroblast of Yorkshire. HH, adult pig ear fibroblast. P, passage.
